# P-741. Doxycycline Postexposure Prophylaxis Utilization at Ryan White Clinic in New Orleans, Louisiana

**DOI:** 10.1093/ofid/ofaf695.952

**Published:** 2026-01-11

**Authors:** Tat Yau, Mason Calico, Catherine Fontenot, Victoria McCray, Meredith E Clement

**Affiliations:** LSU Health New Orleans School of Medicine, New Orleans, LA; LSU Health New Orleans, New Orleans, Louisiana; LSU Health New Orleans, New Orleans, Louisiana; LSU Health New Orleans, New Orleans, Louisiana; Louisiana State University Health Science Center–New Orleans, New Orleans, LA

## Abstract

**Background:**

In June 2024, the Centers for Disease Control and Prevention (CDC) recommended that gay, bisexual, and other men who have sex with men (MSM), as well as transgender women (TGW) who have had a bacterial sexually transmitted infection (STI) diagnosed in the past 12 months receive counseling from their providers using a shared decision-making approach regarding the use of doxycycline postexposure prophylaxis (doxy PEP) for STI prevention.

The HIV Outpatient Program (HOP) at University Medical Center New Orleans provides care to people with HIV (PWH) in the Greater New Orleans area. In 2024, HOP served a total of 1,685 clients, among whom 531 (35%) acquired HIV through MSM, and 27 (1.6%) identified as TGW. The purpose of this study was to evaluate the utilization of doxy PEP among eligible clients at HOP.

**Table 1:**
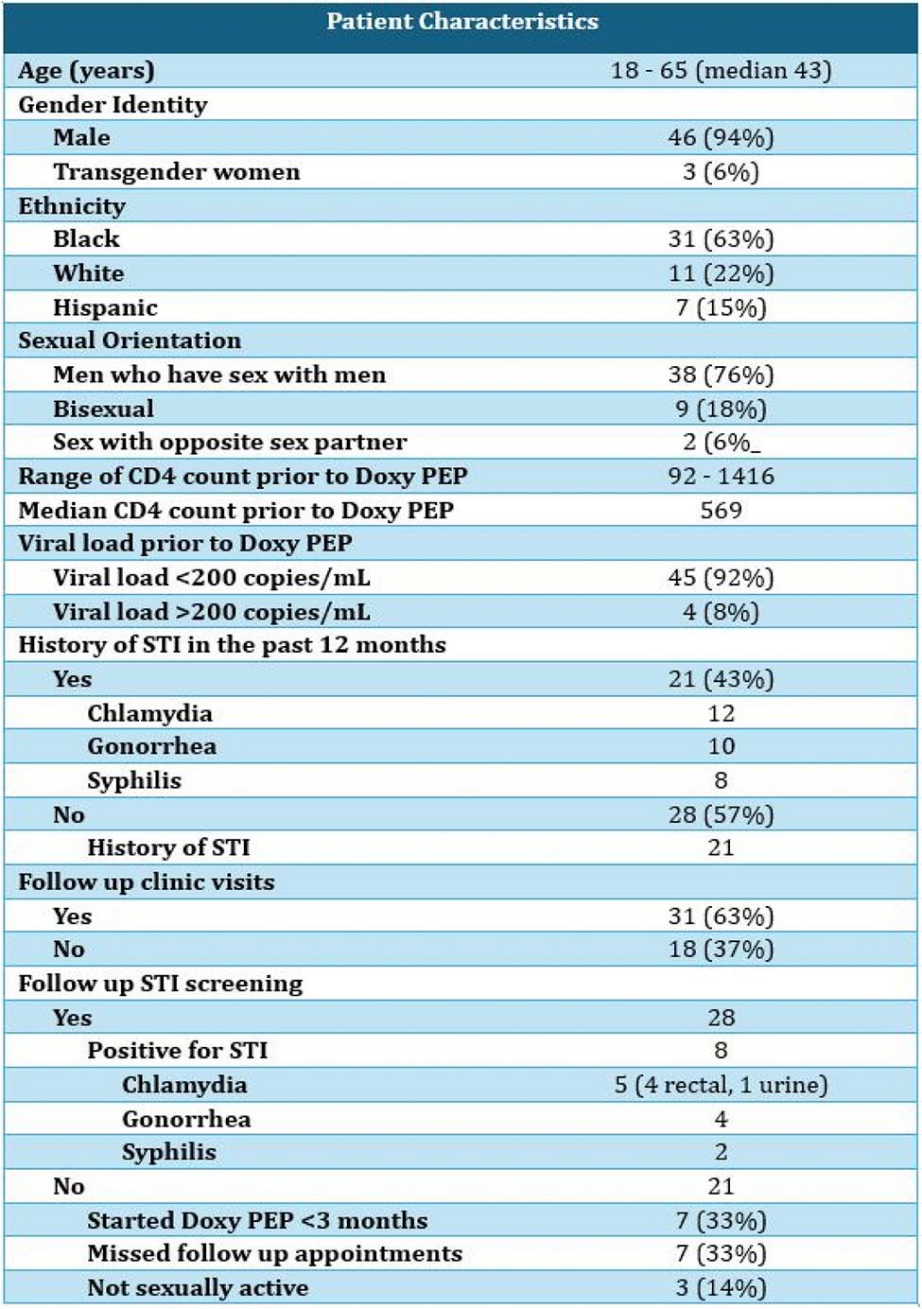
Characteristics of our patients who received doxy PEP from January 2024 to March 2025.

**Methods:**

We extracted data from electronic medical records of PWH prescribed doxycycline in our clinic from January 1, 2024, to March 31, 2025. Charts were reviewed to confirm doxycycline indication and to document demographic characteristics, HIV viral load (VL), CD4 count, and diagnoses of bacterial STIs both prior to and following doxy PEP prescription.

**Results:**

During the study period, a total of 49 eligible patients received prescriptions and were followed for median follow up of 4 months. See Table 1 for baseline characteristics. Participants were aged 18 to 65 years, and 46 were cisgender men and 3 were TGW. The median CD4 count was 569, and 45 (92%) were virally suppressed. Among these patients, 21 (43%) had a history of bacterial STI within the 12 months preceding doxy PEP, while another 21 (43%) reported history of STI diagnosis. 31 patients had follow-up visits, of whom 28 of them had follow-up STI testing. There were 8 patients tested positive for a STI. Among 21 patients who have not had follow-up STI screening, 7 (33%) missed their appointment, 7 (33%) were not yet due for follow-up, and 3 (14%) reported that they were no longer sexually active.

**Conclusion:**

Doxy PEP is being utilized at an HIV clinic in the Deep South of the United States, although uptake may still be low. STIs diagnoses on doxy PEP were higher than would be expected, but additional follow up is needed, and further assessments of adherence and antimicrobial resistance are warranted.

**Disclosures:**

Meredith E. Clement, MD, Gilead Sciences Inc: Grant/Research Support|Viiv Healthcare: Advisor/Consultant|Viiv Healthcare: Grant/Research Support|Viiv Healthcare: Speaker

